# Current use of pain scores in Dutch ICUs: a postal survey in the Netherlands

**DOI:** 10.1186/cc10939

**Published:** 2012-03-20

**Authors:** M Van der Woude, L Bormans, J Hofhuis, P Spronk

**Affiliations:** 1 Atrium Medical Center, Heerlen, the Netherlands; 2 Gelre Hospitals, Apeldoorn, the Netherlands

## Introduction

Pain is a common problem for patients admitted to the ICU, causing patient discomfort, agitation and accidental self-extubation. For this reason the recognition of pain and its severity is extremely important. Several pain scores and protocols are in use. We aimed to elucidate current practice of pain measurements and treatment in Dutch ICUs.

## Methods

In March 2011, a questionnaire was sent to all Dutch adult ICUs irrespective of the number of ICU beds with active follow-up by telephone calls to optimize the participation rate.

## Results

A total of 84 ICUs (84/107) returned the survey, representing a response rate of 87%. Most ICUs are community teaching hospitals and nonteaching hospitals (85%) in comparison to academic hospitals (15%). Most ICUs (94%) use a standardized pain score in the group of patients who are capable of verbal communication: the Visual Analogue Scale (57%), Numerical Rating Scale (48%) and Faces Pain Scale (5%) being the most frequently used scores. In the group of patients who are unable to communicate, ICUs less frequently use pain scores (19%), with the Critical-Care Pain Observation Tool (6%) and Behaviour Pain Scale (5%) being used most frequently. Measurement of pain was considered most important for patients with burn wounds (67%), trauma patients (64%), postoperative patients (57%) and those who receive end-of-life care (64%). Barriers to use pain measurements included the patient's inability to communicate (82%), interference with pain assessment due to sedation (79%), hemodynamic instability (64%), insufficient dosages of analgesics (60%) and the unavailability of a standard pain scoring system (51%). In addition, guidelines for management of sedation and analgesics from the Netherlands Association for Intensive Care (NVIC) had been read by only 20% of the respondents. Factors that were mentioned to be useful in contributing to an improvement in pain assessment and effective pain control included adequate analgesic dosage (87%), utilization of protocols and directives (86%), enthusiastic and motivated personnel (81%) and the utilization of standardized pain measurement tools.

## Conclusion

Most Dutch ICUs measure pain frequently (94%) in patients who are able to communicate. However, in the group of patients who cannot communicate only 19% of the Dutch ICUs use a standardized pain score. This finding applied to both academic and nonacademic ICUs, which suggests that efforts should be put into implementing pain measures in Dutch ICUs.

